# Diet, Gut Microbiome, and Microbial Metabolites in Inflammatory Bowel Disease: From Functional Dysbiosis to Precision Nutrition

**DOI:** 10.3390/ijms27125262

**Published:** 2026-06-10

**Authors:** Josko Bozic, Roko Santic, Piero Marin Zivkovic, Marko Kumric

**Affiliations:** 1 Department of Pathophysiology, University of Split School of Medicine, 21000 Split, Croatia; roko.santic@mefst.hr (R.S.); marko.kumric@mefst.hr (M.K.); 2 Laboratory for Cardiometabolic Research, University of Split School of Medicine, Soltanska 2A, 21000 Split, Croatia; 3 Gastroenterology and Hepatology Department, University Hospital of Split, Spinciceva 1, 21000 Split, Croatia; piero.zivkovic@gmail.com

**Keywords:** inflammatory bowel disease, Crohn’s disease, ulcerative colitis, gut microbiome, diet, microbial metabolites, epithelial barrier, mucosal immunity, functional dysbiosis, precision nutrition

## Abstract

Inflammatory bowel disease (IBD; Crohn’s disease and ulcerative colitis) arises from convergent dysfunction of the epithelial barrier, mucosal immunity, and gut microbiome on a background of genetic susceptibility and environmental exposures. Diet is among the most modifiable of these exposures, yet much of the diet–microbiome research in IBD remains descriptive and poorly aligned with the molecular pathways linking food to mucosal effects. This comprehensive review reframes the field around functional dysbiosis, in which altered microbial metabolic capacity (rather than taxonomic shifts alone) drives disease-relevant biology. We trace how dietary substrates and additives are converted by gut microbes into bioactive metabolites (short-chain fatty acids, secondary bile acids, tryptophan-derived indoles, sulfur compounds, and polyphenol-derived molecules) and map these to host receptors and signaling pathways governing barrier function, mucus and antimicrobial peptide production, and Treg/Th17 balance. Defined dietary therapies (exclusive enteral nutrition, the Crohn’s disease exclusion diet plus partial enteral nutrition, and Mediterranean-style patterns) are reinterpreted as interventions that reshape microbial metabolic output, and candidate biomarkers for microbiome-informed precision nutrition are evaluated. Microbiota-derived metabolites provide the molecular interface between diet and mucosal immunity in IBD; personalized dietary algorithms remain a research goal, not a validated clinical tool, and diet is best framed as adjunctive to pharmacotherapy and dietitian care.

## 1. Introduction

Although diet is increasingly recognized as a modifiable therapeutic factor in inflammatory bowel disease (IBD), the field remains limited by a mismatch between clinical dietary categories and the molecular pathways through which food-derived substrates are transformed by the microbiome into immunologically active metabolites. IBD, encompassing Crohn’s disease (CD) and ulcerative colitis (UC), reflects a self-amplifying loop of genetic susceptibility, epithelial barrier dysfunction, immune dysregulation, microbial alteration, and environmental exposures that converges on chronic relapsing intestinal inflammation [1,2,3]. Among environmental exposures, diet is unique because it is continuous, modifiable, and biochemically intimate: every meal reshapes the substrate pool of the colon and, with it, the metabolic output of the resident microbiota [4,5].

In this review, precision nutrition is defined operationally as the use of individual disease phenotype, nutritional status, habitual diet, inflammatory biomarkers, and, when validated, microbiome or metabolomic features to select, adapt, and monitor dietary strategies for a specific patient. This definition does not imply that microbiome-stratified dietary algorithms are currently ready for routine IBD care; rather, it provides a translational framework for moving from population-level dietary advice toward testable patient-level hypotheses.

Two practical problems have constrained progress. First, much of the diet–microbiome literature in IBD continues to focus on coarse taxonomic shifts, loss of obligate anaerobes, expansion of *Proteobacteria*, reduction in butyrate producers, even though longitudinal multi-omic data show that disease activity is more tightly aligned with functional and metabolomic shifts than with composition alone [6,7]. Second, dietary advice in IBD is often framed in terms of food groups rather than the metabolic pathways that link those foods to mucosal biology. The result is a translational gap between what patients are told to eat and the biochemical levers that diet actually pulls.

This review proposes that the gap is best closed by treating microbiota-derived metabolites as the central organizing concept. In this framework, dietary exposures are upstream ecological and biochemical pressures; the microbiome is a metabolic converter; metabolites such as short-chain fatty acids (SCFAs), secondary bile acids, tryptophan-derived indoles, sulfur compounds, and polyphenol-derived molecules are the signaling output; and host receptors, including G-protein-coupled receptors (FFAR2/3, GPR109A), the bile acid receptors farnesoid X receptor (FXR) and Takeda G-protein-coupled receptor 5 (TGR5), and the aryl hydrocarbon receptor (AhR), are the biological effectors that govern barrier integrity and mucosal immunity [8,9,10,11,12,13,14]. Recent guidance from the European Society for Clinical Nutrition and Metabolism (ESPEN) and the European Crohn’s and Colitis Organisation (ECCO) increasingly anchors clinical recommendations to this mechanistic substrate [15,16].

Within this framework, defined dietary therapies such as exclusive enteral nutrition (EEN), the Crohn’s disease exclusion diet plus partial enteral nutrition (CDED+PEN), and Mediterranean-style patterns can be reinterpreted as interventions that act, at least in part, by reshaping microbial metabolic output [17,18,19,20]. Conversely, ultra-processed foods, dietary emulsifiers, and selected non-nutritive sweeteners can be framed as exposures that perturb microbial ecology and metabolite balance in directions unfavorable for the inflamed intestine [21,22,23]. This review follows the sequence from dietary exposure to microbial ecology, microbial metabolism, host signaling, epithelial and immune responses, and finally clinical biomarkers and precision nutrition.

The added value of this review is therefore not another catalogue of microbial taxa or dietary prescriptions. Prior metabolite-focused reviews have described SCFAs, bile acids, indoles, and related pathways in IBD, whereas diet-focused reviews have summarized EEN, CDED, Mediterranean-style patterns, and exclusion diets. Here, we integrate those studies into a single exposure-to-metabolite-to-host-signaling sequence, explicitly separating clinical evidence from mechanistic plausibility and identifying where microbiome-informed precision nutrition is ready for practice, plausible as an adjunctive strategy, or still research-only.

## 2. Literature Search and Review Approach

This article is a narrative review informed by a structured literature search; it is not a systematic review or a scoping review. The review was not prospectively registered, no formal PRISMA flow diagram was generated, and no quantitative risk-of-bias grading was performed, because the aim was conceptual synthesis of diet–microbiome–metabolite mechanisms and translational readiness rather than exhaustive evidence mapping.

PubMed/MEDLINE, Scopus, Web of Science and Google Scholar were searched between 3 April and 29 April 2026, with additional screening of major guideline and consensus documents from ECCO, ESPEN, ESPGHAN, and IOIBD. Representative search strings included: (“inflammatory bowel disease” OR “Crohn’s disease” OR “ulcerative colitis”) AND (diet OR nutrition OR “exclusive enteral nutrition” OR “Crohn’s disease exclusion diet” OR Mediterranean OR “low-FODMAP” OR “specific carbohydrate diet” OR CD-TREAT OR UCED OR “IBD-AID” OR emulsifier OR “ultra-processed food”); and (“inflammatory bowel disease” OR Crohn’s disease OR “ulcerative colitis”) AND (microbiome OR microbiota OR dysbiosis OR metabolomics OR “short-chain fatty acid” OR butyrate OR “bile acid” OR tryptophan OR indole OR “hydrogen sulfide” OR polyphenol OR “barrier function” OR “mucosal immunity”).

Inclusion criteria were English-language human studies, randomized controlled trials, prospective cohorts, systematic reviews, major guidelines or consensus statements, and mechanistic animal or in vitro studies that directly addressed diet, microbial metabolism, mucosal immunity, epithelial barrier biology, or clinically relevant dietary strategies in IBD. Studies were excluded or de-prioritized when they were not IBD-specific, did not address diet or microbial metabolic pathways, were based only on weakly connected mechanisms, or duplicated evidence already covered by higher-level sources.

Screening was iterative. Titles, abstracts, and full texts identified from database searches and reference lists were reviewed by the author team, with priority given to: (i) human interventional evidence with objective inflammatory or endoscopic endpoints; (ii) prospective human cohort data; (iii) systematic reviews and guidelines; and (iv) mechanistic studies used only to explain biological plausibility. Because this was a narrative synthesis rather than a systematic-style review, a formal number of records considered is not reported.

Levels of evidence were considered when interpreting mechanistic and clinical findings. Because diet–microbiome research in IBD spans different levels of evidence, causal language was used conservatively throughout this review. In vitro and animal studies were interpreted as evidence of mechanistic plausibility; associative human cohort, epidemiological, and multi-omic studies were interpreted as hypothesis-generating and insufficient to establish causality; and interventional human studies were interpreted according to disease phenotype, age group, intervention duration, comparator, and clinical endpoint. Validated clinical application was reserved for dietary strategies supported by clinical trials and/or guideline recommendations. Microbiome, metabolomic, permeability, cytokine, transcriptomic, and sulfur-related markers are therefore described as candidate biomarkers or research tools unless externally validated for patient-level dietary decision-making.

## 3. Functional Dysbiosis in IBD: Definition and Measurement

The classical description of dysbiosis in IBD is taxonomic. Across cross-sectional cohorts, the inflamed intestine shows reduced microbial diversity, depletion of obligate anaerobes within *Lachnospiraceae* and *Ruminococcaceae* (notably *Faecalibacterium prausnitzii* and *Roseburia* spp.), and relative expansion of facultative anaerobes such as *Enterobacteriaceae*, including *Escherichia coli* pathovars [24,25]. In CD, the adherent-invasive *E. coli* (AIEC) pathovar is enriched in the ileal mucosa of a subset of patients and contributes mechanistically to mucosal injury [26,27]. These taxonomic patterns are reproducible and clinically informative, but they are insufficient as a mechanistic framework. Patients with similar 16S rRNA profiles can have very different metabolic outputs, and treatments such as EEN can drive remission while paradoxically reducing the relative abundance of taxa generally considered beneficial.

Multi-omic studies have therefore reframed dysbiosis as primarily functional. The longitudinal Integrative Human Microbiome Project showed that periods of disease activity in CD and UC are characterized by coordinated shifts in metagenomic gene content, microbial transcription, and the fecal metabolome, including reductions in SCFAs and secondary bile acids and rises in conjugated primary bile acids and acylcarnitines [6,7]. Targeted metabolomic and metatranscriptomic work has confirmed that the inflamed gut harbors a microbial community whose metabolic capacity is altered (reduced butyrogenesis, impaired 7α-dehydroxylation of bile acids, decreased microbial production of AhR-active indoles, increased proteolytic and sulfur fermentation, and impaired polyphenol transformation) even when its species composition is only modestly perturbed [28,29,30,31].

Functional dysbiosis in IBD can therefore be defined as a measurable shift in microbial metabolic output, rather than taxonomic composition alone, away from homeostatic signaling and toward a profile associated with epithelial barrier and mucosal immune imbalance [5,31,32]. For the purposes of this section, these alterations are presented as operational features and measurable readouts rather than as receptor-level mechanisms. The main measurable features include reduced SCFA output, particularly butyrate [8,9,33,34]; altered bile acid pools with relative depletion of secondary bile acids [10,11,35,36,37]; reduced microbial production of tryptophan-derived indoles/AhR ligands [12,13,14,38]; increased proteolytic and sulfur-fermentation signatures [39,40,41]; and altered polyphenol-derived metabolite output [42,43,44]. The downstream receptor-level and immune consequences of these metabolite classes are discussed in Section 5.

This framing has two practical implications. First, taxonomic profiles alone are insufficient because clinical response can occur with marked compositional shifts; EEN is illustrative, as clinical improvement and mucosal healing may coincide with reduced microbial diversity and changes in taxa generally considered beneficial [45,46]; second, functional dysbiosis is best captured by pathway- and metabolite-level readouts—SCFA panels, bile acid profiles, indole/AhR-ligand profiles, sulfur/proteolytic signatures, and metagenomic or metatranscriptomic pathway data—that complement, rather than duplicate, taxonomic data. Mucosa-associated microbiota provide an additional layer: the community in direct contact with the inflamed epithelium is more dysbiotic than the luminal community in many CD patients, and its functional output likely matters more for pathogenesis than fecal stool sequencing alone can resolve [28,47].

Two additional caveats are important. Causality remains an open question for many of the changes summarized above: dysbiosis is at once a partial driver and a partial consequence of inflammation, and longitudinal multi-omic data show coordinated bidirectional shifts during dysbiotic episodes [6]. Inter-individual variation also dominates over disease effects in many comparisons, which limits the diagnostic value of any single microbial or metabolite feature considered in isolation. Functional dysbiosis is therefore best understood not as a single pattern, but as a family of overlapping deviations whose composition varies between patients, between disease subtypes, and over time. Functional dysbiosis should also be viewed as one component of a broader exposome model, rather than as the sole explanation for IBD emergence. The hygiene/”old friends” and biodiversity hypotheses propose that reduced early-life and ongoing contact with diverse environmental microbiota may impair immune tolerance and narrow host-associated microbial diversity, offering a complementary explanation for rising IBD incidence that is not reducible to diet alone [48,49]. In parallel, diet may signal directly to the host because dietary and digestion-derived ligands can engage AhR and nutrient-sensing GPCRs/FFARs on epithelial and immune cells; thus, microbiota-derived metabolites are treated here as a central interface, but not the only diet-to-immune pathway [50,51,52]. Accordingly, this section defines and operationalizes functional dysbiosis as a measurable construct, whereas Section 5 addresses the molecular pathways through which major microbiota-derived metabolite classes affect epithelial and immune biology.

## 4. Dietary Exposures Shaping Microbial Metabolism in IBD

### 4.1. Western Diet, Ultra-Processed Foods and Food Additives

Western dietary patterns, high in saturated and ω-6 polyunsaturated fats, refined carbohydrates, and animal protein, and low in fermentable fibers and polyphenols, have been associated in prospective cohorts with increased risk of incident CD and, less consistently, UC [4,5]. In a large prospective cohort, Narula et al. associated higher ultra-processed food (UPF) intake with increased risk of incident IBD [21], and analyses of the Nurses’ Health Study and Health Professionals Follow-up Study reported a stronger signal for incident Crohn’s disease than for ulcerative colitis [47]. These associations are epidemiological and do not prove causation; the consistency across populations and the dose–response signal nonetheless motivate a focus on the bioactive components of UPFs that interact with the microbiome.

Among these, synthetic emulsifiers have received the most mechanistic attention. In murine models, low-dose carboxymethylcellulose (CMC) and polysorbate 80 erode the inner mucus layer, allow microbial encroachment onto the epithelium, increase pro-inflammatory flagellin expression in the microbiota, and exacerbate colitis in genetically susceptible hosts [53]. Ex vivo studies of human microbiota in mucosal simulators reproduce these effects, and a randomized controlled-feeding trial of CMC in healthy adults showed reduced microbial diversity, altered metabolomic profile, and microbial encroachment on the mucosa [22,54]. A pilot study in CD demonstrated that emulsifier restriction is feasible and tolerable [55]. Other additives, including selected carrageenans, gums, and maltodextrin, reproduce parts of this phenotype in vitro, although their relative contribution in humans is uncertain [56,57]. Human evidence supports an association between UPF-rich dietary patterns and incident IBD risk, while mechanistic and early clinical studies point to mucus disruption, epithelial permeability and pathobiont expansion as plausible disease-relevant pathways [21,22,53]; reduction in UPF and additive intake is therefore reasonable as part of general healthy dietary counseling in IBD, but it has not been established as a stand-alone anti-inflammatory therapy for active disease [16].

Non-nutritive sweeteners and additional UPF components illustrate the limits of mechanistic certainty. Personalized human studies show that responses to non-nutritive sweeteners are highly variable and microbiota-dependent, with some individuals exhibiting glycemic and microbial perturbation while others do not [58]. Western-diet patterns also expand AIEC niches in CD-prone hosts and amplify innate immune activation through TLR/NF-κB signaling [26,27]. Taken together, the human evidence supports an association between UPF-rich Western diets and IBD risk and activity, while mechanistic work points to the mucus layer, epithelial permeability, and pathobiont expansion as proximate targets [56,59]. However, the UPF–IBD association should be interpreted cautiously: most epidemiological data rely on self-reported dietary assessment, UPF classification captures heterogeneous products, and higher UPF intake may partly proxy for smoking, physical activity, socioeconomic position, medication use, and other Western-lifestyle factors. Signals are also not fully uniform across phenotypes or exposure definitions, with available cohorts suggesting stronger and more reproducible associations for CD than for UC, while microbiome mediation analyses may be distorted by host variables such as bowel transit, stool consistency, alcohol intake, antibiotic exposure, and disease activity. We therefore frame UPF/additive reduction as reasonable dietetic counseling supported by convergent epidemiology and mechanistic plausibility, not as proof that UPFs alone causally initiate or perpetuate IBD [21,47,56,60].

### 4.2. Fiber, Resistant Starch, Prebiotics and Fermentable Substrates

Fermentable dietary fiber and resistant starch are the substrates from which colonic SCFAs are produced. Long-term high-fiber intake is associated with greater abundance of SCFA producers and with butyrate-rich enterotypes, while short-term dietary shifts can reproducibly remodel community structure within days [61,62]. In IBD, lower habitual fiber intake correlates with higher disease activity and incidence in some prospective cohorts, particularly in CD, and fiber-fortified or prebiotic interventions have shown modest improvements in symptom and inflammatory markers in selected populations [4,63]. Current ECCO and ESPEN guidance treats fiber as conditionally beneficial rather than universally so, and explicitly advises caution in stricturing CD and during active inflammation [15,16].

Mechanistically, fermentable substrates supply colonocytes with butyrate as their primary energy source, support tight-junction integrity, sustain mucus production by goblet cells, and provide ligands for SCFA-sensing receptors that promote regulatory T-cell (Treg) differentiation [8,33,64]. Importantly, the relationship between fiber and IBD outcomes is not uniformly favorable. In stricturing CD or in patients with active inflammation and partial obstruction, high-fiber diets can precipitate symptomatic worsening and bowel obstruction, and abrupt fiber loading can transiently trigger gas, bloating, and visceral hypersensitivity. Preclinical work indicates that the effect of fermentable fiber depends on the baseline host–microbiota context: in selected dysbiotic mouse models, specific fermentable fibers can exacerbate intestinal inflammation through microbiota fermentation-NLRP3 signaling [65], and separate preclinical work has linked dysregulated soluble-fiber fermentation in dysbiotic mice to cholestatic liver injury and hepatocellular carcinogenesis [66]. Fiber and prebiotic strategies therefore require patient selection, particularly in stricturing Crohn’s disease or obstructive symptoms; active symptoms alone should not be equated with a universal indication for fiber restriction, and most patients in quiescent or non-stricturing disease tolerate or benefit from fiber within a varied dietary pattern.

### 4.3. Protein Source and Microbial Nitrogen Metabolism

The protein composition of the diet shapes both the substrate available for microbial nitrogen metabolism and the supply of specific amino acids, most notably tryptophan and the sulfur-containing amino acids cysteine and methionine, that drive immunologically active downstream pathways [39]. When fermentable carbohydrate availability is limited, animal-protein-rich dietary patterns can favor proteolytic fermentation, increasing amino-acid-derived luminal metabolites such as ammonia, branched-chain fatty acids, phenols and p-cresol [39]. Animal-protein-rich Western patterns have been associated with greater UC incidence in prospective cohorts, although the magnitude of effect is modest and confounded by overall dietary pattern [4,5].

Plant-forward protein patterns, legumes, nuts, seeds, and whole grains alongside moderate fish and poultry, provide protein within a substrate matrix rich in fermentable fiber and polyphenols, which buffers proteolytic fermentation and supports SCFA production. In Mediterranean-style cohorts, this combined dietary architecture is associated with lower IBD incidence and with favorable metabolomic profiles [63,67]. Within protein-quality considerations, dietary tryptophan is a focal substrate: its microbial transformation into indole, indole-3-acetate, indole-3-aldehyde, indole-3-propionate, and related ligands feeds the AhR pathway that maintains epithelial repair and IL-22-mediated antimicrobial defense [12,68]. Glutamine, the principal energy substrate for enterocytes, supports barrier function, although clinical trials of glutamine supplementation in IBD have produced mixed results [15].

Sulfur-amino-acid intake represents a parallel axis. High dietary sulfate from sulfur-rich amino acids and certain processed-food preservatives feeds sulfate-reducing bacteria (SRB) such as *Desulfovibrio piger*, increasing colonic hydrogen sulfide [40,41]. In susceptible hosts, this can disrupt mucin disulfide bonding and impair colonocyte mitochondrial respiration. The clinical implication is not that protein should be restricted in IBD, malnutrition and sarcopenia are far more common and clinically dangerous than excess protein intake [15], but that protein source and matrix matter for microbial metabolic output. Plant-forward protein substitution may be particularly relevant for Western-pattern, animal-protein-rich diets, while sulfur-related biomarkers should be evaluated in research settings before being used for routine patient-level dietary stratification.

### 4.4. Dietary Fats and Bile Acid Remodeling

Dietary fat shapes microbial ecology through the bile acid pool. The amount and composition of fat ingested determines the magnitude of hepatic bile acid secretion and the relative output of glycine- versus taurine-conjugated bile acids. High saturated milk-fat intake can increase taurine-conjugated bile acid availability and favor sulfur-utilizing, bile-tolerant pathobionts, classically *Bilophila wadsworthia* in IL-10-deficient mouse models [69]. In humans with CD, the bile acid pool is contracted and shifted toward conjugated primary bile acids with relative depletion of secondary bile acids such as deoxycholate (DCA) and lithocholate (LCA) [35,36]. This shift is driven by reduced abundance of bile-salt hydrolase (BSH)-positive and 7α-dehydroxylating consortia, and it occurs early in dysbiotic episodes.

Beyond a simplistic ω-3 versus ω-6 narrative, lipid quality matters because it shapes the substrate–community–metabolite triangle. Diets rich in long-chain saturated fats favor sulfur-using and bile-tolerant taxa; diets rich in monounsaturated fats and ω-3 polyunsaturated fats are associated with more diverse SCFA-producing communities and with anti-inflammatory eicosanoid profiles [63].

### 4.5. Polyphenols, Fermented Foods and Bioactive Food Components

Most dietary polyphenols are poorly absorbed in the small intestine and arrive in the colon, where the microbiota transforms them into bioactive metabolites including urolithins (from ellagitannins), equol (from isoflavones), and a range of phenolic acids [42]. Urolithin A in particular has been shown to enhance mitophagy, support tight-junction protein expression, and exert AhR/Nrf2-dependent anti-inflammatory effects in preclinical models of colitis [43,44]. Importantly, urolithin-producing capacity is highly variable between individuals: only a subset of adults harbor the *Gordonibacter* and related taxa that complete the conversion of ellagic acid to urolithin A, illustrating that polyphenol benefits are inherently microbiome-dependent and personal [42,70].

Fermented foods are biologically plausible modulators of mucosal homeostasis through delivery of live microbes, exopolysaccharides, and bioactive peptides, but high-quality evidence in IBD remains limited. A 17-week randomized dietary intervention in healthy adults reported that a fermented-food-rich pattern increased microbial diversity and decreased multiple markers of systemic inflammation, whereas a high-fiber arm in the same trial produced more variable individual responses [71]. Extrapolation of these findings to active CD or UC is not justified; within current evidence, fermented foods may be reasonable as components of a Mediterranean-style maintenance pattern but should not be presented as anti-inflammatory therapy in IBD.

### 4.6. Defined Dietary Therapies in IBD

Defined dietary therapies are now part of IBD care in selected settings, but the strength and clinical meaning of evidence differ substantially by age group, disease phenotype, treatment phase, and endpoint [15,16]. The following summary therefore distinguishes pediatric from adult evidence, CD from UC, induction from maintenance, symptom response from biochemical response, and mucosal healing from clinical remission.

The strongest evidence is in pediatric active luminal CD, where ECCO-ESPGHAN guidance recommends EEN as first-line induction therapy and trials show high clinical remission rates with meaningful mucosal-healing responses in a subset of patients [16,72,73]. Adult EEN evidence is less robust and implementation is constrained by adherence, palatability, social burden, and dietitian capacity [72]. EEN has been associated with reproducible microbiome and metabolome shifts, sometimes including lower microbial diversity despite clinical improvement [45,46]. These microbial changes should be interpreted as candidate mediators or response markers rather than proof of causality. EEN is not established as an induction strategy for UC outside selected nutritional-support contexts. EEN reduces dietary diversity, eliminates exposure to many UPF additives, and produces reproducible shifts in fecal microbiome composition and function, often with a paradoxical further reduction in microbial diversity that coincides with biochemical and clinical improvement [45]. EEN has also been associated with shifts in hydrogen sulfide-producing taxa and in proteolytic fermentation, but whether these microbial changes are causal mediators of the anti-inflammatory effect or downstream markers of substrate withdrawal remains uncertain [16,45]. Adherence is the principal limitation, particularly in adolescents and adults [72].

Crohn’s disease exclusion diet plus partial enteral nutrition (CDED+PEN) was designed to recapitulate EEN’s substrate-withdrawal logic in CD while permitting a structured whole-food diet. Pediatric induction evidence is the strongest: a randomized controlled trial in pediatric CD showed that CDED+PEN was better tolerated than EEN and produced higher rates of sustained corticosteroid-free remission at week 12, with reductions in fecal *Proteobacteria* tracking clinical response [17]. Adult evidence has expanded with a 2025 randomized controlled trial of CDED in adults with CD, complementing the earlier open-label adult CDED pilot [74,75]. Pediatric maintenance evidence has also been updated: a 2025 randomized controlled trial reports that a modified CDED maintains remission in pediatric Crohn’s disease [76]. CDED+PEN has been studied specifically in CD; it is not an established induction strategy in UC [16].

Mediterranean-style dietary patterns are characterized by high consumption of fruits, vegetables, whole grains, legumes, nuts, olive oil, and fish, with limited red and processed meat. In adults with CD and mild-to-moderate symptoms, the Mediterranean diet was comparable to the Specific Carbohydrate Diet for symptomatic remission at 6 weeks in the DINE-CD trial, with similar improvements in fatigue and quality of life but no clear superiority over the comparator on objective markers of inflammation [18]. Prospective cohort data link higher adherence to the Mediterranean pattern with a lower risk of later-onset CD [67]. The Mediterranean pattern is a reasonable maintenance and cardiometabolic strategy in IBD; it has not been established as a stand-alone induction therapy for active intestinal inflammation [16,77]. Key human trials underlying these distinctions are summarized in Table 1, including study design, sample size, disease phenotype, duration, endpoints, and narrative evidence level.

Low-FODMAP diets reduce intake of fermentable oligo-, di-, and monosaccharides and polyols, a concept originally developed and validated in irritable bowel syndrome [78]. In quiescent IBD with persistent IBS-like symptoms, IBD-specific randomized trials show symptomatic improvement without measurable changes in objective markers of intestinal inflammation [79,80,81]. Low-FODMAP diets may therefore reduce IBS-like or functional gastrointestinal symptoms in quiescent IBD but should not be presented as induction therapy, maintenance therapy, or anti-inflammatory dietary therapy for active IBD [16]; prolonged restriction can reduce microbial diversity and SCFA production and should be supervised by a dietitian.

UPF and additive reduction, and plant-forward protein substitution, are pragmatic strategies grounded in the mechanisms reviewed above [47,56]. They align with general healthy-eating recommendations endorsed by ECCO and IOIBD [15,16] but, given the largely epidemiological evidence base, should not be presented as established anti-inflammatory treatments for active IBD. Across all defined therapies, two cautions are essential: restrictive diets carry real risks of malnutrition, micronutrient deficiency, and disordered eating patterns [15,16]; and dietary therapy should complement, not replace, evidence-based pharmacotherapy with corticosteroids, immunomodulators, biologics, or small molecules [82].

UC-focused exclusion approaches remain early-stage. The UC exclusion diet (UCED), with or without antibiotic rescue, has been evaluated in a small open-label pediatric pilot and in the CRAFT UC trial context, but these data do not yet establish UCED as routine UC induction therapy [83,84]. A low-fat, high-fiber diet improved quality of life and markers of inflammation/dysbiosis in a small UC crossover trial, supporting further study but not broad disease-modifying claims [85].

IBD-AID is supported mainly by retrospective case-series data and remains an adjunctive, hypothesis-generating strategy rather than a validated treatment [86]. Low-emulsifier or additive-restriction approaches are biologically plausible and feasible in CD but remain early clinical concepts [55,56]. Plant-based or semi-vegetarian maintenance strategies are attractive because they reduce Western-diet exposures and increase fiber/polyphenol substrate; however, the best-known semi-vegetarian CD study was single-center and requires replication before it can be treated as established maintenance therapy [87].

Across all defined therapies, restrictive diets carry real risks of malnutrition, micronutrient deficiency, sarcopenia, social burden, and disordered eating patterns [15,16]. Dietary therapy should therefore complement, not replace, evidence-based pharmacotherapy with corticosteroids, immunomodulators, biologics, or small molecules [82], and should be delivered with dietitian oversight whenever restriction is substantial.

Table 2 summarizes the major dietary exposures, microbial metabolic pathways, host signaling mechanisms and translational implications discussed in this review.

## 5. Microbiota-Derived Metabolites as Molecular Mediators

**Figure 1:**
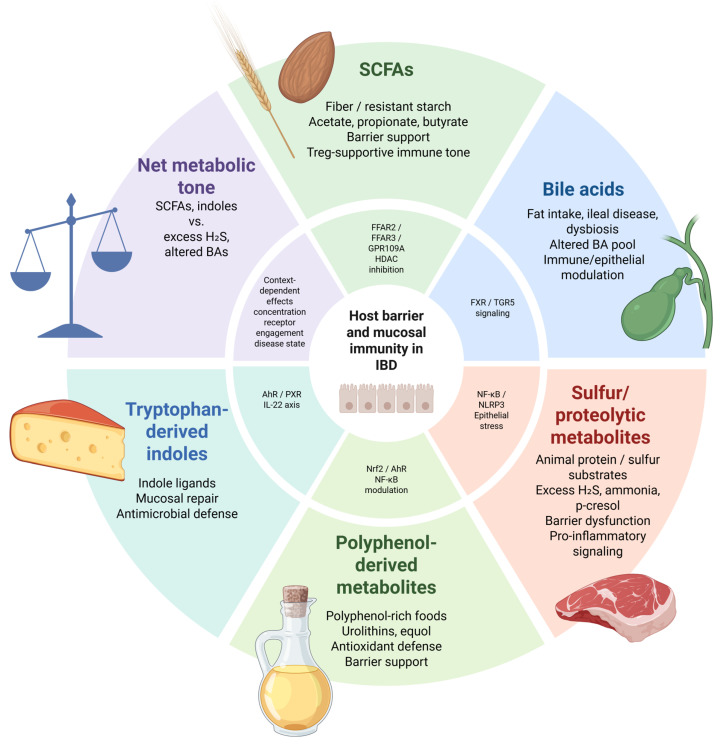
Microbiota-derived metabolite classes linking diet to epithelial barrier function and mucosal immunity in IBD. Dietary substrates are transformed by the gut microbiome into bioactive metabolite classes that regulate epithelial and immune pathways. SCFAs, tryptophan-derived indoles, polyphenol-derived metabolites and selected bile acid signals generally support barrier integrity, antimicrobial defense, epithelial repair and regulatory immune tone through pathways including FFAR2/FFAR3, GPR109A, HDAC inhibition, AhR/PXR–IL-22 signaling, Nrf2 and FXR/TGR5. In contrast, excessive sulfur and proteolytic fermentation may increase H_2_S, ammonia and p-cresol exposure, promoting epithelial stress, barrier dysfunction and pro-inflammatory signaling. The net mucosal effect depends on metabolite balance, concentration, receptor engagement and disease context. Created in BioRender. Kumric, M. (2026) https://BioRender.com/9nodkez (accessed on 4 May 2026).

Having defined functional dysbiosis as a measurable shift in microbial metabolic output, the dietary exposures and defined therapies reviewed above converge on a common intermediate step: altered substrate availability to the microbiota, which reshapes metabolite signals reaching epithelial and immune pathways. The principal microbiota-derived metabolite classes linking dietary exposures to epithelial barrier function and mucosal immunity in IBD are summarized in Figure 1. Their biological effects depend on metabolite concentration, receptor engagement, disease state, and the net balance of microbial metabolic output.

### 5.1. Short-Chain Fatty Acids as Diet-Derived Regulators of Barrier Function and Treg Responses

Acetate, propionate, and butyrate are produced by anaerobic fermentation of dietary fiber and resistant starch, and together they reach 50–100 mM concentrations in the healthy colon. Butyrate is produced principally by *Faecalibacterium prausnitzii*, *Roseburia* spp. and members of the *Lachnospiraceae*; in IBD, these taxa are depleted and luminal butyrate concentrations decline, particularly during disease activity [6,25,64]. The biological consequences are multilayered.

First, butyrate is the preferred energy substrate for colonocytes. In germ-free mice, deficiency of microbially produced butyrate triggers a cell-autonomous shift to autophagy and impairs epithelial energy metabolism, with collapse of tight-junction integrity reversed by butyrate replacement [33]. Second, SCFAs signal through G-protein-coupled receptors (FFAR2/GPR43, FFAR3/GPR41, and GPR109A) that are expressed on colonocytes, enteroendocrine cells, and innate immune cells. Through GPR43 and GPR109A, SCFAs facilitate dietary-fiber-induced gut homeostasis, restrain inflammasome activity in epithelial cells, and promote IL-10 production [8,9]. Third, butyrate inhibits histone deacetylases (HDAC), particularly histone deacetylase 3, in colonic regulatory T cells, expanding peripherally induced Foxp3+ Tregs and biasing the mucosal Treg/Th17 axis toward tolerance [34,89]. Fourth, butyrate supports goblet-cell mucin (MUC2) production and Paneth-cell antimicrobial peptide expression, indirectly limiting microbial encroachment on the epithelium [64,90].

Translationally, restoring SCFA tone in IBD remains conceptually appealing, but the therapeutic relevance of direct butyrate supplementation depends strongly on formulation and delivery site. Earlier experience with unformulated oral butyrate and topical butyrate enemas produced inconsistent or modest benefits, partly because proximal absorption and segment-limited exposure can prevent sustained delivery to the inflamed colon. Newer delayed-release, colon-targeted, and microencapsulated sodium-butyrate formulations are designed to overcome this limitation, and recent randomized data in mild-to-moderate UC, pediatric IBD, and mixed adult IBD cohorts report improvements in clinical activity, fecal calprotectin and, in UC, endoscopic improvement when used as an adjunct to conventional therapy [91,92,93]. These data justify a more nuanced view of exogenous SCFA delivery, but they do not yet establish butyrate supplementation as a stand-alone anti-inflammatory therapy or a substitute for dietary strategies that restore SCFA-producing ecology, such as high-fiber Mediterranean-style patterns, EEN-induced microbial remodeling, and CDED+PEN [17,18].

### 5.2. Bile Acid Remodeling at the Interface of Dietary Fat, Microbial Metabolism and FXR/TGR5 Signaling

For readability, bile acid signaling is discussed here in three steps: dietary fat-driven bile acid secretion, microbial conversion of primary into secondary bile acids, and host FXR/TGR5-mediated effects on epithelial and immune signaling. Hepatic primary bile acids, including cholate and chenodeoxycholate, are conjugated to glycine or taurine and secreted into the duodenum, where they emulsify lipids. Most are reabsorbed in the terminal ileum, but a fraction reaches the colon, where the microbiota performs two key transformations: deconjugation by bile-salt hydrolases (BSH), expressed broadly across *Lactobacillaceae*, *Bifidobacteriaceae*, *Bacteroidaceae*, and *Clostridia*, and 7α-dehydroxylation by a narrower consortium of *Clostridium scindens*-related species, generating the secondary bile acids deoxycholate (DCA) and lithocholate (LCA) [10,94].

In CD and to a lesser extent UC, this transformation is impaired: BSH and 7α-dehydroxylating capacity drop, glycine- and taurine-conjugated primary bile acids accumulate, and DCA/LCA become depleted [35,36,95]. The receptor consequences are clinically meaningful. FXR, the major nuclear receptor for primary bile acids, restrains bacterial translocation, induces antimicrobial peptide expression, and suppresses NF-κB-driven epithelial inflammation [11]. TGR5 supports epithelial renewal and regeneration after intestinal injury and contributes to bile-acid-mediated modulation of mucosal inflammation [10,96].

Microbially produced 3-oxoLCA and isoalloLCA shift the mucosal Treg/Th17 balance: 3-oxoLCA inhibits Th17 differentiation while isoalloLCA expands RORγ+ peripheral Tregs [97,98].

Therapeutic implications follow from this framework. Strategies that aim to restore secondary bile acid output, either by repopulating BSH and 7α-dehydroxylating taxa, by supplying defined consortia of bile acid-modifying organisms, or by targeting FXR/TGR5 pharmacologically, are an active area of translational interest. In preclinical humanized mouse models, gut microbiota-related bile acid metabolism through the FXR/TGR5 axis has been implicated in response to anti-α4β7-integrin therapy [88].

Whether bile acid signatures can serve as validated predictors of biologic response in patients with IBD remains unresolved. No FXR or TGR5 modulator is currently approved for IBD, and present dietary strategies act on the upstream substrate ecology of these receptors rather than engaging them directly [10].

### 5.3. Tryptophan-Derived Indoles, AhR Activation and Mucosal Immune Tolerance

Dietary tryptophan can be metabolized along three principal routes: host kynurenine pathways, host serotonin pathways, and microbial indole pathways. In the colon, *Lactobacillus*, *Peptostreptococcus*, *Clostridium sporogenes*, and selected *Bacteroides* species transform tryptophan into indole, indole-3-aldehyde (IAld), indole-3-acetate (IAA), indole-3-propionate (IPA), indole-3-lactate (ILA), and indoleacrylic acid, which serve as ligands for the aryl hydrocarbon receptor (AhR) and, to a lesser extent, the pregnane X receptor (PXR) [12,68]. AhR activation in intestinal lymphoid cells, particularly group 3 innate lymphoid cells (ILC3s) and a subset of intraepithelial lymphocytes, drives IL-22 production, which acts on epithelial cells to induce Reg3-family antimicrobial peptides, promote mucin production, and accelerate epithelial proliferation and repair [9,13].

In IBD, this axis is impaired in two complementary ways. First, microbial production of AhR ligands is reduced, as documented in CARD9-deficient hosts and in human IBD cohorts [38,99]. Second, host kynurenine pathway flux is increased during active inflammation, producing kynurenine and quinolinate that, although AhR-active, are associated with disease activity rather than tolerance [29,99]. The net effect is a redirection of tryptophan from microbial indole production toward host kynurenine metabolism, with attenuation of IL-22-driven epithelial repair.

Translationally, AhR-targeted strategies include increasing dietary tryptophan in protein-replete contexts, providing prebiotic substrates that favor indole-producing taxa, and developing microbially derived or synthetic AhR agonists with selectivity for intestinal pathways [14,44]. The polyphenol-derived metabolite urolithin A engages AhR/Nrf2 cooperativity, suggesting that polyphenol- and tryptophan-derived AhR ligands converge on shared barrier-protective programs [43,44].

### 5.4. Sulfur Metabolites and Proteolytic Fermentation

Hydrogen sulfide (H_2_S) is produced from dietary sulfate, sulfur-containing amino acids, and taurine-conjugated bile acids by sulfate-reducing bacteria, including *Desulfovibrio* spp. as part of the broader sulfur-metabolism axis implicated in IBD [40,41]. Concentrations of H_2_S in the colonic lumen range from micromolar to low millimolar, and its biological effect is concentration-dependent. At low concentrations, H_2_S is an endogenous gasotransmitter that supports colonocyte mitochondrial function and modulates inflammation; at higher concentrations, it inhibits cytochrome c oxidase, depletes mucin disulfide bonding, and disrupts colonocyte energetics [39,100].

Most disease-relevant evidence for sulfur metabolism remains preclinical or associative. In the canonical taurocholate-milk-fat colitis model, high saturated milk-fat intake increases taurine-conjugated bile acid availability and expands the bile-tolerant, taurine-respiring pathobiont *Bilophila wadsworthia* in IL10^−/−^ mice [69]. This model is mechanistically informative, but it does not justify patient-level sulfur restriction without clinical validation.

Other proteolytic and sulfur-derived metabolites contribute to mucosal stress. Ammonia from microbial deamination, p-cresol from tyrosine fermentation, and phenols from aromatic amino acid metabolism each impair colonocyte mitochondrial respiration and have been linked to barrier disruption in animal models [39,40,41,100]. Clinically, the current implication is cautious dietary pattern modification, limiting Western-pattern saturated fat and excess processed meat while preserving adequate protein intake from plant-forward and lean animal sources [16,101]. H_2_S-related markers should be treated as research biomarkers, not routine clinical tools for prescribing low-sulfur diets.

### 5.5. Polyphenol-Derived Metabolites

Polyphenol-derived microbial metabolites integrate the diet–microbiome–barrier axis from a different direction. Urolithins, equol, and phenolic acids (produced from ellagitannins, isoflavones, and flavonoids by colonic communities) activate Nrf2-dependent antioxidant programs, dampen NF-κB-driven inflammation, and induce tight-junction proteins and MUC2 expression [43,70]. Urolithin A enhances mitophagy in epithelial and immune cells, an effect that translates into measurable mitochondrial improvements in human supplementation studies [43]. The clinical caveat is interindividual variability: producer status varies geographically and with habitual diet, and supplementation studies in IBD specifically remain small.

### 5.6. Systemic Metabolite Spillover and Cardiometabolic Implications

Microbial choline, betaine, and L-carnitine metabolism produces trimethylamine, which is hepatically oxidized to trimethylamine-N-oxide (TMAO). TMAO has been linked in non-IBD populations to endothelial dysfunction, foam-cell formation, platelet hyperreactivity, and cardiovascular risk [102]. In the present review, TMAO is not treated as a central mediator of intestinal mucosal inflammation. It is retained as a systemic spillover example because IBD-specific data link TMAO more consistently to dysbiosis, disease-activity markers, and endothelial or coronary microvascular dysfunction than to direct epithelial or mucosal immune injury. Available studies are small, observational, and directionally inconsistent, including reports of reduced circulating TMAO in active UC/CD and Mendelian-randomization data that do not provide clear evidence that genetically predicted TMAO increases IBD [103,104,105,106,107]. TMAO should therefore not be used as a stand-alone dietary decision marker in IBD. Instead, it is best viewed as one example of systemic metabolite spillover, supporting the broader point that dietary management in IBD should also consider extraintestinal cardiometabolic risk rather than focus exclusively on mucosal inflammation. Dysbiosis-related alterations in SCFAs, secondary bile acids, indoles, and lipopolysaccharide spillover provide a broader biochemical bridge between intestinal inflammation and cardiometabolic risk in long-standing IBD [108,109].

## 6. Host Signaling Pathways Linking Microbial Metabolites to Mucosal Inflammation

To improve readability, these host-side effects are grouped into three effector layers: epithelial barrier and mucus defense, innate immune and redox pathways, and adaptive Treg/Th17 regulation. Dietary substrates and microbial metabolites converge on a limited set of host effector compartments, principally the intestinal epithelium, innate immune cells, and adaptive lymphocytes. At the epithelial surface, tight-junction integrity, mediated by claudins, occludin, and zonula occludens-1, is reinforced by butyrate-driven energy supply, FXR/TGR5-dependent epithelial signaling, AhR/Nrf2-induced tight-junction protein expression, and urolithin A-driven mitophagy [65,110]. Loss of these convergent inputs, as occurs during functional dysbiosis, increases paracellular permeability and exposes lamina propria immune cells to luminal antigens [111].

The same metabolite networks also shape the mucus and antimicrobial defense system. MUC2-rich inner mucus is sustained by butyrate and by AhR-dependent goblet-cell programs, whereas emulsifier exposure and H_2_S overload can thin and disorganize the mucus layer, allowing microbial encroachment [39,53]. Paneth-cell function is supported by TGR5 signaling, AhR-driven IL-22, and SCFA-induced HDAC inhibition, which together promote expression of α-defensins, lysozyme, and Reg3 family antimicrobial peptides that limit bacterial translocation [14,90].

Microbial metabolites also regulate innate and adaptive immune tone. SCFAs and bile acid-TGR5 signaling can favor IL-10-producing macrophage phenotypes, whereas excessive sulfide and lipopolysaccharide promote NF-κB- and inflammasome-mediated activation [9,100]. Dendritic cells exposed to SCFAs and bile acid metabolites adopt more tolerogenic profiles, supporting Treg induction in mesenteric lymph nodes [97,112].

At the adaptive immune level, SCFAs through HDAC inhibition and GPR43-mediated signaling, bile acid metabolites such as 3-oxoLCA and isoalloLCA, AhR ligands through IL-22 and ILC3 support, and polyphenol-derived metabolites collectively bias the mucosal immune environment toward Foxp3+ Treg differentiation and IL-10/IL-22-mediated repair, while restraining IL-17A/IL-23-driven inflammatory programs [14,98]. In IBD, functional dysbiosis attenuates these tolerogenic signals and lowers the threshold for Th17- and innate-driven inflammation.

A parallel layer of regulation occurs through epithelial redox biology and innate immune pattern-recognition pathways. Mitochondrial dysfunction, neutrophil-derived reactive oxygen species, and H_2_S-driven respiratory inhibition place stress on the epithelial redox environment [100,113]. Microbial polyphenol metabolites, particularly urolithin A, and tryptophan-derived AhR ligands engage Nrf2-dependent programs that support glutathione synthesis and antioxidant gene expression [43,113]. At the same time, SCFAs, especially butyrate, restrain NLRP3 inflammasome activation, whereas flagellin, lipopolysaccharide, and other microbial products from pathobiont-enriched communities engage TLR4/TLR5-dependent NF-κB signaling and amplify inflammation [26,53].

Two integrative concepts emerge from these pathways.

The first is convergence: distinct microbial metabolites engage overlapping host programs. SCFAs, urolithin A, and tryptophan-derived AhR ligands all support tight-junction protein expression; secondary bile acids, SCFAs, and AhR ligands converge on Treg expansion and Th17 restraint; and AhR, Nrf2, and FFAR signaling jointly maintain epithelial redox balance and antimicrobial peptide production [8,98]. This redundancy can be protective in health, but it also means that no single metabolite is the master regulator of mucosal homeostasis.

The second concept is directionality. In healthy mucosa, microbial metabolite signaling biases the epithelium and immune compartment toward tolerance and repair. In IBD, functional dysbiosis shifts the dominant signaling direction toward pathobiont expansion, NF-κB and inflammasome activation, and Th17-skewed immunity [114,115].

Taken together, these mechanisms indicate that diet–microbiome–metabolite perturbations in IBD do not act through a single pathway, but through coordinated weakening of multiple tolerogenic inputs and amplification of multiple inflammatory inputs. This integrated view helps explain why single-metabolite or single-pathway interventions have generally produced incremental rather than transformational effects, whereas dietary therapies that remodel substrate flow across several axes, including EEN, CDED+PEN, and Mediterranean-pattern adherence, may be more clinically impactful than narrow nutritional supplementation. It also reinforces the complementary roles of pharmacotherapy and nutrition: biologics and small molecules act on host effector pathways, while dietary strategies act upstream on the substrate ecology that shapes the metabolite signals those host pathways receive [15,82].

## 7. Toward Microbiome-Informed Precision Nutrition in IBD

Table 3 summarizes the clinical readiness, proposed mechanisms, limitations and candidate monitoring biomarkers for microbiome-informed dietary strategies in IBD.

Two facts limit one-size-fits-all dietary advice in IBD. First, IBD is biologically heterogeneous: CD and UC differ in location, behavior, and immune signature; CD itself spans inflammatory, stricturing, and penetrating phenotypes; and disease activity, prior surgical history, and concomitant medications further fragment the population. Second, dietary responses are personal: identical foods produce divergent glycemic, microbial, and metabolomic outputs across individuals, with the gut microbiome explaining a meaningful portion of the variance [116,117]. Microbiome-informed precision nutrition aims to use this individuality, rather than ignore it, to design dietary strategies; at present, however, validated, microbiome-stratified dietary algorithms for IBD do not exist, and proof-of-concept work has largely been conducted in non-IBD populations [16,118]. Accordingly, the term precision nutrition is used here to denote an emerging research trajectory rather than an available clinical algorithm. Current practice should remain phenotype-, nutrition-, and inflammation-guided use of established dietary strategies, whereas microbiome- or metabolome-guided dietary prescription should be considered aspirational or research-only until prospectively validated in IBD-specific trials with objective inflammatory and endoscopic endpoints [15,16,116,118,119].

### 7.1. Patient Phenotyping as the Starting Point

Before any microbiome-based personalization, baseline phenotyping must capture: disease type (CD vs. UC), location (ileal, colonic, ileocolonic; left-sided vs. extensive UC), disease behavior (inflammatory, stricturing, penetrating), current disease activity, current medications (especially biologics, JAK inhibitors, sphingosine-1-phosphate modulators, corticosteroids, and recent antibiotics), nutritional status (body mass index, sarcopenia indices, hand-grip strength, micronutrient panels), habitual diet, and prior dietary restrictions [15,119,120]. Without this scaffolding, microbiome data are uninterpretable. ECCO and ESPEN both recommend systematic nutritional screening every 3 months in active disease and annually in remission, including iron, vitamin B12, vitamin D, calcium, and dietary fiber adequacy [15,16].

### 7.2. From Taxonomy to Function: Multi-Omic Substrate for Personalization

Taxonomic 16S rRNA profiles, although accessible, are insufficient for individual-level dietary decision-making in IBD. Metagenomic shotgun sequencing reveals the gene content for SCFA, bile acid, and tryptophan pathways; metatranscriptomics captures which of those genes are expressed during dysbiotic episodes [28,30]. Metabolomics, including targeted SCFA panels, bile acid profiling, and tryptophan/indole panels, measures the actual metabolic output and is more closely tied to clinical state than taxonomy alone [6,7,29]. Genome-scale metabolic reconstructions of the human microbiome now allow in silico prediction of metabolic capacity from sequencing data and represent a candidate scaffold for individualized modeling [121]. Mucosa-associated microbiome sampling, although more invasive, captures the community in direct contact with the epithelium and may better predict response to mucosa-active therapies than stool sampling [28,122].

### 7.3. Inflammation and Barrier Biomarkers

Fecal calprotectin (FC) and CRP should be separated from exploratory biomarkers. FC is the most robust non-invasive biomarker of intestinal inflammation in IBD, with high sensitivity and a high negative predictive value at the 50–100 microg/g cut-off and strong correlation with endoscopic activity, particularly in UC [119,123]. CRP complements FC and is most useful in CD with transmural inflammation, but CRP can be normal despite active mucosal inflammation [124]. FC performance is more variable in isolated small-bowel CD and cut-off dependent [125,126], so confirmation with capsule endoscopy, magnetic resonance enterography, intestinal ultrasound or endoscopy may be required when symptoms and biomarkers are discordant.

By contrast, epithelial permeability markers (zonulin, lactulose/mannitol ratios), serological markers such as anti-AIEC antibodies and anti-Saccharomyces cerevisiae antibodies, cytokine panels (TNF, IL-6, IL-22, IL-23), mucosal transcriptomics, and most metagenomic/metabolomic readouts remain investigational in dietary decision-making [26,27,127,128]. They may be valuable in trials or specialized translational cohorts, but they should not be presented as validated tools for routine diet prescription.

### 7.4. Adherence Biomarkers and Digital Dietary Assessment

A persistent obstacle in dietary research is the unreliability of self-reported intake. Objective biomarkers of adherence, such as urinary alkylresorcinols for whole-grain intake, plasma carotenoids for fruit and vegetable intake, and metabolomic fingerprints of Mediterranean adherence, offer a path to verification [118]. Digital food recording, image-based intake estimation, and continuous glucose and microbiome sensing are converging into multi-modal monitoring platforms suitable for use in clinical trials [117,118]. Adherence biomarkers and standardized digital dietary assessment are also research priorities highlighted in current ECCO dietary guidance [16].

### 7.5. Toward Microbiome-Informed Dietary Algorithms

Several proof-of-concept studies have shown that machine-learning models trained on microbiome and host features can predict postprandial glycemic response, weight-loss trajectories, and microbial responses to specific dietary patterns in healthy and metabolic-disease cohorts [116,118]. Translating this to IBD requires three additional ingredients: (i) IBD-specific endpoints (fecal calprotectin, endoscopic activity, time-to-relapse, biologic response); (ii) longitudinal training data that capture diet, microbiome, metabolome, and clinical course before, during, and after dietary intervention; and (iii) externally validated, interpretable models that clinicians can integrate with disease phenotype and pharmacotherapy decisions. Prospective microbiome and metabolomic studies suggest that baseline microbial composition, microbial functional pathways and fecal or serum metabolic profiles may help predict response to anti-integrin and anti-TNF therapy in IBD; however, such models remain adjunctive research tools and require external validation before clinical deployment [129,130].

Given current evidence, biomarker-informed dietary decision-making in IBD should primarily support the selection, monitoring, and safe implementation of established dietary strategies rather than prescribe microbiome-tailored diets de novo. In routine practice, this means using clinical phenotype, nutritional risk, fecal calprotectin, CRP, and treatment goals to prioritize established approaches such as EEN or CDED+PEN in appropriate CD settings [72,74]; Mediterranean-pattern maintenance in adults in remission or with cardiometabolic risk factors [18,67]. By contrast, scenarios such as tailoring fiber or prebiotic intensification to documented butyrate-producer depletion [6,64] or modifying protein sources according to H_2_S-related markers and Western dietary patterns [69] should be framed as research hypotheses or future clinical scenarios, not as routine patient-level prescriptions. UPF and additive reduction can still be encouraged broadly as general healthy dietary counseling, but not as a stand-alone anti-inflammatory treatment for active IBD [47,56].

These recommendations should be made with appropriate humility. Microbiome-informed precision nutrition in IBD is an active area of investigation but is not yet mature enough for routine algorithmic patient-level dietary prescription. Until prospective trials demonstrate that microbiome-stratified dietary strategies improve hard outcomes, such as mucosal healing, transmural healing, surgery-free survival, and biologic-free remission, precision nutrition should be offered as an adjunct to evidence-based pharmacotherapy and dietitian-led care, not as a replacement for it [15,16].

Operational considerations also constrain near-term translation. Microbiome assays are not yet standardized across reference laboratories; turnaround times, sequencing depth, and bioinformatic pipelines vary widely, with downstream effects on taxonomic and functional readouts [6,117]. Targeted metabolomic panels for SCFAs, secondary bile acids, indoles, and sulfur metabolites are technically feasible but not yet broadly accessible in routine gastroenterology care [29,31]. Reimbursement frameworks for multi-omic profiling remain underdeveloped, and dietitian capacity is limited even in tertiary IBD centers [15]. Pragmatic clinical workflows will therefore require staged implementation: starting with low-cost, high-yield metrics such as fecal calprotectin trajectory, dietary pattern characterization, and structured UPF/additive screening, and reserving deeper microbiome and metabolome interrogation for non-responders, complex phenotypes, and prospective research cohorts [15,16].

## 8. Research Gaps and Future Directions

Specific, actionable gaps persist across the diet–microbiome–metabolite–immune axis in IBD. A first priority is the standardization of dietary assessment. Current trials in IBD use heterogeneous tools, including food diaries, 24-h recalls, and food-frequency questionnaires, which limits cross-study comparability and makes it difficult to connect dietary exposure with microbial and inflammatory outcomes. IBD-specific, validated digital tools that capture both nutrient composition and additive/UPF exposure are needed, ideally paired with objective adherence biomarkers [46,131].

A second major gap is the lack of longitudinal, multi-omic sampling. Most existing diet–microbiome studies in IBD remain cross-sectional and stool-based, which limits causal interpretation and underrepresents the mucosal compartment. Longitudinal studies integrating metagenomics, metatranscriptomics, fecal and plasma metabolomics, mucosal transcriptomics, and immune profiling before, during, and after dietary intervention are required to move from association to mechanism [7,30]. Although mucosa-associated sampling at colonoscopy is invasive, it provides irreplaceable information about the microbial community in direct contact with inflamed tissue and may better capture disease-relevant functional dysbiosis than stool profiling alone [28,122].

Mechanistic validation is also essential. Hypotheses generated by human cohorts must be tested in defined-microbiota gnotobiotic mice, intestinal organoids and organoid-immune co-cultures, and isogenic microbial knockouts that can pinpoint specific metabolic pathways such as BSH activity, 7α-dehydroxylation, and indole production [68,94]. The Debaryomyces work in CD-associated wounds illustrates the value of model systems in identifying unexpected microbial contributors and clarifying how specific organisms or pathways may impair tissue repair [132].

Clinical translation will require pragmatic randomized trials with appropriate stratification. Beyond pediatric EEN and CDED+PEN, there remains a shortage of adequately powered dietary intervention trials in adult IBD, and a near-complete absence of trials that stratify participants by phenotype, disease activity, medication exposure, and baseline microbiome state. Future trials should be designed around real-world clinical questions: which dietary strategy is best for a given disease phenotype, alongside which pharmacologic therapy, and for which endpoint, including symptomatic remission, fecal calprotectin response, mucosal healing, or time to relapse [15,16]. A particularly important trial design space is diet as an adjunct to advanced therapy. Few studies have tested dietary intervention specifically alongside anti-TNF, anti-IL-23, anti-α4β7, JAK inhibitors, or sphingosine-1-phosphate modulators, even though bile acid-FXR/TGR5 data suggest that microbial metabolic state may condition response to biologics [10,82].

Predictive modeling is another promising but immature area. Models intended to predict dietary response in IBD must be developed against IBD-specific endpoints, validated in independent cohorts, and made interpretable enough for clinical integration. Models trained in metabolically healthy or general-population cohorts should not be assumed to transfer directly to IBD, where inflammation, medication exposure, disease location, and mucosal damage alter diet–microbiome relationships; therefore, IBD-specific predictive models built on inflammatory and nutritional endpoints are required [46,118]. Genome-scale metabolic reconstructions of the human gut microbiome offer a complementary approach that is more mechanistically transparent than purely opaque machine-learning models and can be linked to host pathway models for hypothesis generation [121].

Finally, future work should better define how dietary therapy influences systemic disease beyond the gut. Cardiometabolic comorbidity, fatigue, and bone health interact with intestinal dysbiosis in IBD, and several microbiota-derived metabolites, including TMAO, indoxyl sulfate, and p-cresyl sulfate, act on tissues distant from the intestine [102,108]. Future trials should therefore consider co-primary endpoints that span intestinal and extraintestinal outcomes, since dietary strategies are unlikely to be neutral with respect to systemic risk. Nonetheless, this broader perspective should not displace the central therapeutic goal in IBD: improving mucosal healing, reducing flare risk, and supporting biologic-free remission.

## 9. Conclusions

Diet should be viewed as a modifiable upstream determinant of microbial metabolic function in IBD rather than as a nonspecific lifestyle exposure. Dietary substrates and food additives shape the microbial production of SCFAs, secondary bile acids, tryptophan-derived indoles, sulfur compounds and polyphenol-derived metabolites, which in turn regulate epithelial barrier integrity, mucus and antimicrobial defense, and mucosal immune balance. The clinical implication is not that microbiome profiles can currently prescribe individualized diets, but that microbial function and metabolite readouts may help refine the selection, timing and monitoring of established dietary strategies. At present, the most defensible use of microbiome-informed nutrition is to support evidence-based interventions such as EEN, CDED+PEN, Mediterranean-style dietary patterns, UPF reduction and symptom-directed low-FODMAP approaches in appropriately selected patients. Future progress will depend on trials that connect dietary exposure, microbial metabolic function, host receptor engagement and clinically meaningful endpoints such as fecal calprotectin response, mucosal healing and relapse prevention.

## Figures and Tables

**Table 1:** Key human dietary intervention studies discussed in Section 4.6.

Dietary Strategy/Study	Study Context	Main Endpoint and Evidence Interpretation
EEN; Pigneur et al. [45]	Pediatric active CD; randomized trial; n = 19; 8 weeks	Clinical remission and mucosal healing. Supports EEN as pediatric CD induction therapy, although trial size was small.
CDED+PEN; Levine et al. [17]	Pediatric mild-to-moderate CD; randomized trial; n = 78; 12 weeks	Corticosteroid-free remission, CRP, fecal calprotectin. Strong pediatric CD induction evidence; not established for UC.
Adult CDED; Yanai et al./Pasta et al. [74,75]	Adult mild-to-moderate CD; pilot/open-label randomized trials; n = 44 and n = 45; up to 24 weeks	Clinical remission and inflammatory markers. Promising adult CD evidence, but still emerging and requiring larger validation.
Modified CDED maintenance; Sigall Boneh et al. [76]	Pediatric CD in remission/step-down setting; randomized trial; n = 56; 24 weeks	Sustained corticosteroid-free remission. Supports pediatric maintenance/step-down use, but evidence remains less mature than induction data.
Mediterranean diet vs. SCD—DINE-CD; Lewis et al. [18]	Adults with CD and mild-to-moderate symptoms; randomized comparative-effectiveness trial; n = 194; 12 weeks	Symptomatic remission, CRP, fecal calprotectin. Reasonable symptom/maintenance strategy; not established as stand-alone anti-inflammatory induction therapy.

CD, Crohn’s disease; CDED, Crohn’s disease exclusion diet; CRP, C-reactive protein; EEN, exclusive enteral nutrition; PEN, partial enteral nutrition; SCD, Specific Carbohydrate Diet; UC, ulcerative colitis. Evidence interpretation is narrative and does not represent formal guideline grading.

**Table 2:** Dietary Exposures, Microbial Mechanisms, and Evidence Categories in IBD.

Dietary Exposure	Main Microbial Effect	Key Metabolite or Pathway	Host Receptor/Signal Pathway	Barrier and Immune Effect	Evidence Category/Interpretation
Fermentable fiber/resistant starch	Saccharolytic shift; ↑ SCFA producers	Acetate, propionate, butyrate (SCFAs)	FFAR2/3, GPR109A; HDAC inhibition	↑ barrier/Treg; ↓ inflammasome tone	Adjunctive/maintenance use if tolerated; avoid stricturing or obstructive CD [15,16,63]
Western diet/UPF	↓ diversity; ↑ facultative anaerobes/AIEC	↓ SCFAs; ↑ lipopolysaccharide; altered BAs	TLR4–NF-κB; ↓ FFAR signaling	Mucus thinning; ↑ permeability	Adjunctive healthy-diet counseling; not active-IBD monotherapy [21,47]
Synthetic emulsifiers	Mucus encroachment; ↑ flagellin	Bioactive flagellin	TLR5-NF-Κb	Mucus disruption; low-grade inflammation;	Research-only/early clinical concept; emulsifier restriction is feasible but not validated as therapy [22,55]
Animal protein/sulfur-rich substrates	↑ *Bilophila*/SRB; proteolysis	H_2_S, ammonia, p-cresol, branched-chain fatty acids	Mitochondrial stress; mucin disruption	Context-dependent barrier effects	Adjunctive dietary counseling; sulfur-related biomarker use remains research-only [15,39,69]
Plant-forward protein patterns	↑ saccharolysis; favorable BA ecology	SCFAs; indoles	FFAR/GPR109A; AhR	Barrier support; tolerogenic milieu	Adjunctive maintenance and cardiometabolic-risk strategy [63,67]
Dietary fat/bile acid remodeling	Altered BSH and 7α-dehydroxylation	Secondary and conjugated BAs	FXR; TGR5; RORγt	Antimicrobial and immune modulation	Research-only/mechanistic; not validated for dietary response prediction [10,88]
Tryptophan-containing substrates	Microbial indole production	IAld, IAA, IPA, ILA	AhR/PXR; IL-22	Antimicrobial peptides, mucus, epithelial repair	Research-only/mechanistic; AhR-targeted dietary strategies remain investigational. [12,13]
Polyphenols	Microbial conversion varies	Urolithins, equol, phenolic acids	AhR; Nrf2; NF-κB inhibition	Mitophagy; tight-junction proteins; antioxidant defense	Research-only/mechanistic; supplementation in IBD remains investigational [42,43]
Exclusive enteral nutrition (EEN)/CDED+PEN	Reduced microbial diversity; reduced UPF/additive exposure; shifts in sulfide producers	EEN-linked metabolome signatures	FFAR, FXR/TGR5, AhR	Mucosal healing and biomarker improvement in pediatric CD	Established clinical use in selected CD contexts: EEN for pediatric CD induction; CDED+PEN for CD, not UC [16,17,72,73,74,75,76]
Mediterranean-style diet	↑ SCFA producers; balanced bile acid pool; rich polyphenol substrate	SCFAs; secondary bile acids; urolithins; phenolic acids	Convergent FFAR/FXR/TGR5/AhR/Nrf2 engagement	Maintained barrier; tolerogenic immune milieu; cardiometabolic benefit	Adjunctive/maintenance use; not stand-alone induction therapy [18,67]

Abbreviations: AhR, aryl hydrocarbon receptor; AIEC, adherent-invasive *Escherichia coli*; BAs, bile acids; CD, Crohn’s disease; CDED, Crohn’s disease exclusion diet; EEN, exclusive enteral nutrition; FFAR, free fatty acid receptor; FXR, farnesoid X receptor; HDAC, histone deacetylase; IAld, indole-3-aldehyde; IAA, indole-3-acetate; ILA, indole-3-lactate; IPA, indole-3-propionate; NF-κB, nuclear factor κB; PEN, partial enteral nutrition; PXR, pregnane X receptor; SCFA, short-chain fatty acid; TGR5, Takeda G-protein-coupled receptor 5; TLR, Toll-like receptor; Treg, regulatory T cell; UC, ulcerative colitis; UPF, ultra-processed food. References in the table are representative and were selected to support the main mechanism or clinical interpretation for each row. Evidence categories indicate the current translational status of each exposure or pathway and should not be interpreted as formal guideline grades. “Research-only” denotes preclinical, associative, or insufficiently validated evidence for patient-level dietary prescription.

**Table 3:** Clinical Readiness of Microbiome-Informed Dietary Strategies in IBD.

Strategy	Best-Supported Context	Clinical Use Category	Evidence Strength	Key Limitations	Biomarkers to Monitor
Exclusive enteral nutrition (EEN)	Induction in pediatric mild–moderate CD; selected adult CD	Established clinical use	Strong in pediatric CD (RCTs and guidelines [16,72,73])	Adherence; psychosocial burden; relapse on resumption of free diet	Fecal calprotectin; CRP; weight; albumin; PCDAI/HBI
CDED + partial enteral nutrition	Induction and maintenance in pediatric and selected adult mild–moderate CD	Established/defined use in CD; strongest pediatric evidence, emerging adult evidence	Strong in pediatrics (induction RCT [17]; maintenance RCT [76]); emerging in adults [74,75]	Whole-food adherence; provider expertise; not established in UC	Fecal calprotectin; CRP; PCDAI/HBI; microbiome shifts
Mediterranean-style diet	Maintenance; cardiometabolic risk reduction; possibly mild–moderate CD symptoms	Adjunctive/maintenance use	Moderate (cohorts; DINE-CD comparator [18,67])	Not established as anti-inflammatory induction therapy; heterogeneous adherence	FC; CRP; lipid panel; quality-of-life indices
Low-FODMAP diet	IBS-like or functional GI symptoms in quiescent IBD; not induction or maintenance therapy for active IBD	Symptom-directed use	Moderate for symptom relief in IBD-specific RCTs [79,80,81]; not anti-inflammatory	Reduced microbial diversity with prolonged restriction; restrictive; should be dietitian-supervised	Symptom scores; FC (to exclude missed inflammation)
Fiber/prebiotic intensification	Quiescent UC; non-stricturing CD	Adjunctive use if tolerated; microbiome-guided use remains research-only	Modest; heterogeneous [63,65]	Tolerability in active disease; obstruction risk in stricturing CD	FC; symptom diaries; SCFA panel (research)
UPF/additive reduction	Most patients with IBD	Adjunctive healthy-diet counseling	Low to moderate; strongest for incident IBD risk and mechanistic plausibility, limited for treatment of established inflammation [21,22,56]	Largely epidemiological evidence; not stand-alone anti-inflammatory therapy for active IBD	FC; dietary adherence biomarkers
Plant-forward protein substitution	Maintenance; cardiometabolic risk reduction	Adjunctive/cardiometabolic strategy; H_2_S-guided use remains research-only	Indirect/inferential [39,69]	Protein adequacy in malnutrition/sarcopenia	Albumin; muscle mass; FC; sulfide-related biomarkers (research)

Abbreviations: CD, Crohn’s disease; CDED, Crohn’s disease exclusion diet; CRP, C-reactive protein; FC, fecal calprotectin; FODMAP, fermentable oligo-, di-, monosaccharides and polyols; HBI, Harvey-Bradshaw Index; IBD, inflammatory bowel disease; IBS, irritable bowel syndrome; PCDAI, Pediatric Crohn’s Disease Activity Index; RCT, randomized controlled trial; SCFA, short-chain fatty acid; UC, ulcerative colitis; UPF, ultra-processed food. Note on biomarkers: FC and CRP are clinically actionable markers; SCFA, bile acid, indole, sulfide and metagenomic/metabolomic profiles remain primarily research tools for dietary stratification and mechanistic phenotyping, not validated clinical decision aids. References in the table are representative and were selected to support the main mechanism or clinical interpretation for each row. Clinical-use categories are intended to separate current practice from adjunctive counseling, symptom-directed strategies, and research-only applications. They do not replace formal guideline grading and should be interpreted in the context of disease phenotype, activity, nutritional status, and dietitian supervision.

## Data Availability

No new data were created or analyzed in this study. Data sharing is not applicable to this article.

## Abbreviations

The following abbreviations are used repeatedly in this manuscript:

3-oxoLCA	3-oxo-lithocholic acid
AhR	aryl hydrocarbon receptor
AIEC	adherent-invasive *Escherichia coli*
BA/BAs	bile acid/bile acids
BSH	bile salt hydrolase
CDED	Crohn’s disease exclusion diet
CDED+PEN	Crohn’s disease exclusion diet plus partial enteral nutrition
CMC	carboxymethylcellulose
DCA	deoxycholic acid/deoxycholate
DINE-CD	Diet to Induce Remission in Crohn’s Disease
ECCO	European Crohn’s and Colitis Organisation
EEN	exclusive enteral nutrition
ESPEN	European Society for Clinical Nutrition and Metabolism
ESPGHAN	European Society for Paediatric Gastroenterology, Hepatology and Nutrition
FC	fecal calprotectin
FFAR/FFAR2/3	free fatty acid receptor/free fatty acid receptors 2 and 3
FODMAP	fermentable oligo-, di-, monosaccharides and polyols
FXR	farnesoid X receptor
GPR41/GPR43/GPR109A	G-protein-coupled receptors 41, 43 and 109A
HBI	Harvey-Bradshaw Index
HDAC	histone deacetylase
IAA	indole-3-acetate
IAld	indole-3-aldehyde
ILA	indole-3-lactate
ILC3	group 3 innate lymphoid cell
IOIBD	International Organization for the Study of Inflammatory Bowel Diseases
IPA	indole-3-propionate
isoalloLCA	isoallolithocholic acid
JAK	Janus kinase
LCA	lithocholic acid/lithocholate
MUC2	mucin 2
NLRP3	NOD-like receptor pyrin domain-containing 3
Nrf2	nuclear factor erythroid 2-related factor 2
PCDAI	Pediatric Crohn’s Disease Activity Index
PEN	partial enteral nutrition
PXR	pregnane X receptor
SCD	Specific Carbohydrate Diet
SCFA/SCFAs	short-chain fatty acid/short-chain fatty acids
SRB	sulfate-reducing bacteria
TGR5	Takeda G-protein-coupled receptor 5
TLR	Toll-like receptor
TMAO	trimethylamine-N-oxide
Treg	regulatory T cell
UPF/UPFs	ultra-processed food/ultra-processed foods
α4β7	alpha-4 beta-7 integrin
